# Infants′ Sunlight Exposure Practice and Associated Factors Among Mothers in Northwest Ethiopia: A Community‐Based Cross‐Sectional Study Community‐Based Cross‐Sectional Study

**DOI:** 10.1002/hsr2.71461

**Published:** 2025-11-06

**Authors:** Tesfaye Shumet Mekonnen, Eneyew Talie Fenta, Anley Shiferaw Enawgaw, Fassikaw Kebede Bizuneh, Amare Mebrat Delie

**Affiliations:** ^1^ Department of Public Health College of Health Sciences, Debre Markos University Debre Markos Ethiopia; ^2^ Department of Public Health College of Health Science, Injibara University Agew Awi Zone Ethiopia

**Keywords:** associated factors, Ethiopia, good practice, infant, sunlight exposure

## Abstract

**Background and Aim:**

Sunlight is crucial for infant health, as it contributes to bone development, immune function, and overall well‐being. However, Maternal sunlight exposure practices are influenced by various sociodemographic and healthcare‐related factors, including maternal age, education level, and access to postnatal care. This study aimed to assess sunlight exposure practices and associated factors among mothers in Gindewoin town.

**Methods:**

A community‐based cross‐sectional study included 397 randomly selected mothers with infants. Data were collected through structured interviews, entered into EpiData version 4.2, and analyzed using SPSS version 26. Binary logistic regression was performed to identify determinants of good sunlight exposure practices.

**Result:**

Only 38% of mothers reported practicing adequate sunlight exposure for their infants. Mothers aged 25–29 years (AOR = 3.01) and 30–34 years (AOR = 4.11) were significantly more likely to practice good sunlight exposure. Diploma and above education increased the odds of good practice by threefold. Lack of postnatal follow‐up was associated with a 74% reduction in the likelihood of adequate sunlight exposure.

**Conclusion:**

The prevalence of good sunlight exposure practice remains low. Interventions should target maternal education, encourage postnatal care utilization, and promote awareness among younger mothers to enhance infant sunlight exposure practices.

**Abbreviations**
ANC: Antenatal Care
AOR: Adjusted Odds Ratio
CI: Confidence Interval
COR: Crude Odds Ratio
PNC: Postnatal Care
SD: Standard Deviation
SPSS: Statistical Package for the Social Sciences
UVB: Ultraviolet B radiation
WHO: World Health Organization.

## 1 Introduction

Infancy is a critical period marked by rapid growth and development, making the role of environmental influences, such as sunlight exposure, particularly crucial [1]. Sunlight serves as a primary natural and cost effective means of obtaining vitamin D [2, 3]. Vitamin D is a vital nutrient essential for bone health, immune system function, and overall well‐being [4, 5, 6, 7]. The practice of ensuring adequate sunlight exposure for infants is, however, contingent upon a myriad of factors deeply embedded in cultural and socioeconomic contexts [8].

According to a World Health Organization (WHO) report, the prevalence of vitamin D deficiency (VDD) in children, specifically infants, ranges from 30% to 80%. It is an important public health problem in both developed and underdeveloped countries and will cause many health related problems including rickets [4, 9]. Infants, in particular, rely heavily on external sources of vitamin D, as their skin is more sensitive and they may not consume enough through diet alone [10, 11]. The duration, timing, and quality of sunlight exposure during infancy can significantly impact a child′s health outcomes [12].

Mothers, as primary caregivers, play a central role in determining infants′ exposure to sunlight [13]. Maternal knowledge, attitudes, and practices regarding sunlight exposure are crucial in shaping the health outcomes of their infants [14]. Understanding the factors that influence mothers′ decisions related to sunlight exposure for their infants is vital for developing targeted interventions and educational programs [15].

Ethiopia′s multicultural society, comprising various ethnic groups and communities, adds complexity to the study of infants′ sunlight exposure practices [16]. Practices related to clothing, outdoor activities, and beliefs about the effects of sunlight on health may differ significantly among these groups, influencing the duration and quality of sunlight exposure for infants [17].

Additionally, access to healthcare services and information about the importance of sunlight exposure can vary across different regions of Ethiopia [16]. Factors such as maternal education, awareness of nutritional requirements, and engagement with healthcare providers can all impact the decisions mothers make regarding infants′ sunlight exposure [18, 19].

Based on the previous studies conducted in Ethiopia, the magnitude of good practice of sunlight exposure ranges from 44% to 58% in various regions [14, 18, 20, 21, 22, 23]. For instance, a study in Dessie Town reported that 41% of mothers exposed their infants to sunlight adequately, whereas in Yirgalem, the proportion was higher, around 54.3% [20, 23]. This variability highlights the presence of regional, cultural, and healthcare disparities affecting maternal practices. According to studies, different factors like maternal and husband education, mother occupation, family size, poor living conditions and unfavorable weather conditions were determining factors of infants′ sunlight exposure [24]. Additionally, fear of cold, fear of evil eyes, and fear of witchcraft were reported as a cause of inadequate sunlight exposure in some studies [14].

Despite the known benefits of sunlight exposure, there is a growing concern about inadequate exposure among infants in various communities, including Ethiopia [10, 16]. This issue is multifaceted and influenced by a range of factors [25]. This includes cultural beliefs and practices, urbanization, lifestyle changes, and prevailing health recommendations [26, 27]. Cultural norms related to clothing practices, perceptions of sunlight as a health risk, and urban living conditions contribute to the variability in infants′ sunlight exposure practices [28].

In light of these considerations, this study was designed as a community‐based cross‐sectional investigation to assess the level of infant sunlight exposure practices and to identify the sociodemographic and healthcare‐related factors influencing these practices among mothers in northwestern Ethiopia. The cross‐sectional design was selected to capture a snapshot of current maternal behaviors and associated factors across a representative sample. This approach allows for quantifying the magnitude of good sunlight exposure practices and exploring potential determinants in the population. Ultimately, the findings aim to inform targeted public health strategies that promote optimal infant sunlight exposure and improve maternal awareness in the local context.

## 2 Methods

### 2.1 Study Area and Period

The study was conducted from June 30, 2024, to July 30/2024 in Gindewoin town mothers, which is located in Goncha district, northwestern Ethiopia. Gindewoin town is located at 335 km far from Addis Ababa, the capital city of Ethiopia and at 150 km far from Bahir Dar, the city of Amhara regional state [29]. The town comprises 7) kebeles (smallest administrative unit in Ethiopia).

### 2.2 Study Design

Community based cross sectional study design was employed.

### 2.3 Source Population

The source population for this study consisted of all mothers with infants below the age of 12 months residing in Gindewoin town. The study included mothers with infants under the age of 12 months who were both living in Gindewoin town and available during the data collection period. This inclusion criterion was implemented to ensure that the sample represented the target population of interest—mothers with young infants—and those who were accessible and willing to participate in the study during the data collection phase.

### 2.4 Eligibility

The study included mothers with infants less than 12 months old who were both mentally and physically capable of providing responses. Exclusion criteria involved mothers who were not regular residents, defined as those living in the town for less than 6 months. These criteria aimed to ensure that the study focused on mothers with a stable and consistent presence in the town, while also selecting participants whose infants were within a specific age range to align with the study objectives.

### 2.5 Sample Size

The sample size was determined using the single population proportion formula, assuming a proportion of poor sunlight exposure practice at 44% (*p* = 0.44). This proportion is derived from a previous cross‐sectional study conducted in Dejen district [30]. The calculation was performed with a 95% confidence level (Z a/2 = 1.96) and an estimated margin of error of 5%. Taking into account a 5% nonresponse rate, the final total sample size required for the study is determined to be 397 mothers.

### 2.6 Sampling Procedure

The research utilized a systematic random sampling approach for participant selection. Mothers who had given birth within the past year were selected from health extension workers associated with each health post. Out of 1327 mothers with infants during the study period, 397 participants were chosen. The initial respondent was selected through a lottery, and thereafter, every third mother (with a constant interval of *k* = 3) from their respective households was interviewed systematically at regular intervals.

### 2.7 Variables and Their Measurement

The outcome variable was the mother′s practice of infant sunlight exposure, assessed using seven structured questions adapted from validated tools in previous studies. Each question was scored as 1 for good practice and 0 for poor practice, resulting in a total possible score ranging from 0 to 7. Mothers who scored equal to or above the sample mean were categorized as having good sunlight exposure practices, while those who scored below the mean were categorized as having poor practices. The questions assessed whether the mother exposed her infant to sunlight, the age at which sunlight exposure began (with earlier initiation, particularly before 6 months, considered favorable), the duration of each exposure session (with at least 15 min regarded as good practice), and the frequency of exposure (with daily exposure being preferred). The questionnaire also evaluated the time of day when exposure occurred, with morning hours between 8:00 and 10:00 AM considered optimal for vitamin D synthesis. Additionally, mothers were asked about the body parts exposed during sunning, where exposing the arms and legs or more was considered beneficial. Lastly, the use of lubricants such as butter or oils before sun exposure was assessed, with non‐use being considered a better practice due to potential interference with UVB absorption The socio‐demographic characteristics of respondents (age, religion, marital status, occupation, family income, educational status, family size), the source of information about sunlight exposure, and pregnancy and delivery‐related characteristics of mothers were assessed as independent variables in the study.

### 2.8 Data Collecting Tools and Procedures

A structured interviewer‐administered questionnaire was used for data collection. The questionnaire was developed based on an extensive review of previous studies and related literature on infant sunlight exposure practices in similar settings [13, 17, 21, 23, 31]. The tool was initially developed in English, translated into the local language (Amharic), and then back‐translated into English to ensure consistency. The questionnaire encompassed socio‐demographic, personal, environmental, and other pertinent variables. Trained Bachelor of Science (BSc) Nurses, along with supervision by one Master of Science (MSc) nurse, conducted face‐to‐face interviews with mothers and infants to collect the data. A day of training was provided to the data collectors, focusing on the study′s objectives, questionnaire content, and issues related to respondent confidentiality and rights. To prevent information contamination, a 5% pretest from the total sample size was conducted in neighboring town, located 27 km away from Gindewoin town.

## 3 Method of Data Analysis

Following data collection, data entry was performed using EpiData version 3.1, and the data set was subsequently exported to the Statistical Package for the Social Sciences (SPSS) version 26 for analysis. Descriptive statistics were used to summarize socio‐demographic variables. Associations between independent and dependent variables were examined using binary logistic regression. In the bivariable analysis, crude odds ratios (COR) with 95% confidence intervals (CI) were calculated to identify candidate variables with a *p*‐value ≤ 0.25, which were then entered into the multivariable logistic regression model. Adjusted odds ratios (AOR) with 95% CI were calculated to control for potential confounders. Variables with a *p*‐value < 0.05 in the multivariable analysis were considered statistically significant factors influencing mothers′ practice of infant sunlight exposure.

## 4 Result

### 4.1 Socio‐Demographic Characteristics

A total of 397 mothers participated in the study, yielding a response rate of 100%. The mean age of the mothers was 27.4 ± 7 years, ranging from 15 to 53 years. More than half of the infants were younger than 4 months, with a mean age of 5 ± 3 months.

Regarding religion, more than half of the participants were Orthodox Christians. A majority (307/397, 77.3%) of the mothers were married. About 16.4% (65/397) of respondents had no formal education, while 37.3% (148/397) had attained a diploma or higher educational status. Most mothers were housewives (139/397, 35%) and merchants (59/397, 14.9%). The average monthly income was 6300 ± 3900 Ethiopian birr, with a range of 800 to 25,000 birr (Table 1).

**Table 1:** Summary of socio demographic characteristics of respondents in Gindewoin town, northwest Ethiopia, 2024.

Demographic variables	Category	Frequency	Percentage (%)
Maternal age	Less than 20 years	58	14.61
	20–24 years	92	23.17
	25–29 years	109	27.5
	30–34 years	64	16.1
	35 and older age	74	18.6
Infant age	One month and younger	32	8.1
	2–5 months of age	210	52.9
	6 month and older	155	39.0
Religion	Orthodox	206	51.9
	Muslim	163	41.1
	Protestant	28	7.1
Marital status	Unmarried	34	8.6
	Married	308	77.5
	Divorced	33	8.3
	Widowed	22	5.5
Maternal education status	Up to grade 12	249	62.72
	College diploma and above	148	37.28
Fathers education (*n* = 308)	Up to grade 12	145	47.1
	College diploma and above	163	52.9
Family income	Less than 2500 birr	72	18.1
	2500–4000 birr	87	21.9
	4001–7500 birr	86	21.7
	More than 7500 birr	152	38.3
Occupation of mother	Student	20	5.0
	House wife	138	34.8
	Government employee	60	15.1
	Private employee	34	8.6
	Daily laborer	48	12.1
	Merchant	97	24.4
Number of children	1–3	297	74.81
	4 and more	100	27.19

### 4.2 Pregnancy‐Related Variables

**Figure 1:**
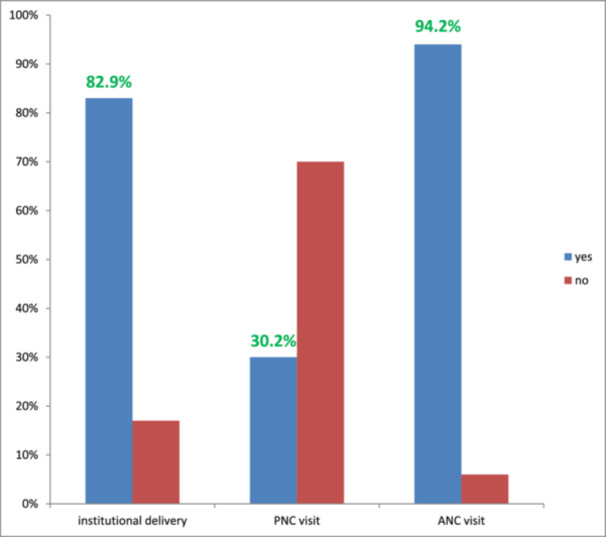
Distribution of pregnancy‐related characteristics among study participants in Gindewoin town, northwest Ethiopia, 2024 (n = 397). The bar chart displays the proportion of respondents who received the service (blue) and those who did not (red) for each pregnancy‐related characteristic assessed. Specifically, institutional delivery (Yes = 82.9%, 95% CI: 79.2%–86.6%), postnatal care (PNC) visit (Yes = 30.2%, 95% CI: 25.7%–34.7%), and antenatal care (ANC) visit (Yes = 94.2%, 95% CI: 91.5%–96.2%) are shown.

Most respondents (327/397, 82.4%) gave birth at a health facility, with 153 mothers delivering at hospitals. Among all respondents, 374 (94.2%) had at least one Antenatal care (ANC) visit. Of those, 96/374 (25.7%) completed the recommended four ANC visits. Only 120/397 (30.2%) mothers reported postnatal care (PNC) follow‐up, with 62/120 (51.7%) having a single Postnatal care (PNC) visit (shown in Figure 1).

### 4.3 Mothers′ Practice of Sunlight Exposure

**Figure 2:**
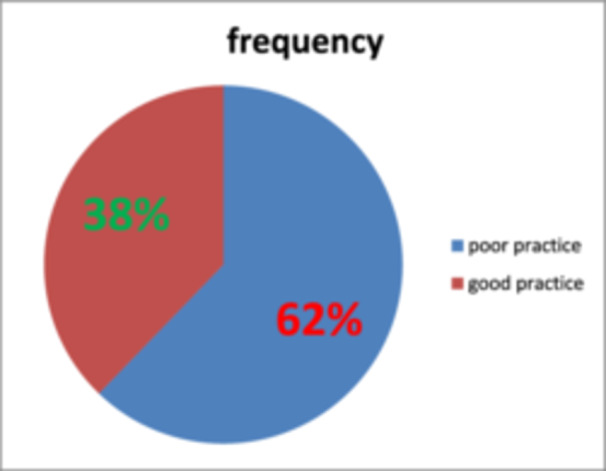
Overall practice of infants′ sunlight exposure among mothers in Gindewoin town, northwest Ethiopia, 2024 (*n* = 397). The pie chart shows the proportion of mothers with good (red) and poor (blue) sunlight exposure practices for their infants. Good practice was assessed using seven practice‐related questions. Among the participants, 38% demonstrated good practice, while 62% had poor practice. Percentages and sample sizes are displayed for each category.

Among the participants, 272/397 (68.5%) mothers reported that they intentionally exposed their infants to sunlight. Of these, 72/272 (26.5%) began sunlight exposure within the first 15 days after birth, and 54/272 (19.9%) started within the first 30 days. The majority (192/272, 70.6%) exposed their infants between 8:00 AM and 10:00 AM. Regarding clothing during exposure, 48/272 (17.6%) exposed infants wore only a diaper, while 132/272 (48.5%) were partially covered. 76/272 (27.9%) exposed their infants daily, and 92/272 (33.8%) did so for 10–15 min per session. Additionally, 146/272 (53.7%) applied lubricants to their infants during exposure (see Table 2). Based on the operational criteria, the overall magnitude of good sunlight exposure practice was 38.0% (151/397; 95% CI: 33.0%–42.5%), as determined by responses to seven practice‐related questions (as shown in Figure 2).

**Table 2:** Mothers practice of their infant′s sunlight exposure in Gindewoin town, northwest Ethiopia, 2024.

Characteristics	Category	Frequency	Percentage
Intentionally expose to sunlight	Yes	270	68.3
	No	127	31.7
Age to start sunning	0–15 days	72	26.6
	15–30 days	54	20.0
	30–45 days	32	11.8
	After 45 days	112	41.5
Condition of clothing	Only diaper	48	17.8
	Partially covered	132	48.9
	Completely covered	90	33.3
Application of lubricants	Yes	146	54.1
	No	124	45.9
Frequency of exposure	Daily	76	28.2
	Sometimes	194	71.8
Duration of exposure	Less than 10 min	6	2.2
	10–15 min	92	34.1
	16–30 min	56	20.7
	More than 30 min	116	43.0
Time of exposure	Morning 8–10 AM	192	71.1
	After noon	78	28.9

### 4.4 Factors Affecting the Practice of Sunlight Exposure

Fifteen variables were initially assessed for their association with mothers′ practice using bivariate logistic regression. Variables with *p* < 0.25 were entered into a multivariable logistic regression model. These included maternal age, marital status, religion, maternal education, place of delivery, family size, number of ANC visits, and PNC follow‐up.

In the final multivariable logistic regression model, four variables remained significantly associated with good sunlight exposure practice (*p* < 0.05). Compared to mothers aged less than 20 years (reference category), those aged 25–29 years were 2.86 times more likely to practice good sunlight exposure (AOR = 2.86; 95% CI: 1.28–6.42; *p* = 0.01), and those aged 30–34 years were 4.15 times more likely (AOR = 4.15; 95% CI: 1.58–10.90; *p* = 0.004). Mothers with a diploma or higher educational level had significantly better practice compared to those with no formal education (reference category), with an AOR of 3.28 (95% CI: 1.88–5.73; *p* < 0.001). Additionally, mothers without postnatal follow‐up were 74% less likely to practice good sunlight exposure than those with postnatal care (AOR = 0.26; 95% CI: 0.15–0.46; *p* < 0.001) (Table 3).

**Table 3:** Bivariate and multivariable analysis of factors associated with good practice of infant′s sunlight exposure among mothers in Gindewoin town, northwest Ethiopia, 2024.

Variables	Category	Good practice (*n* = 149)		Poor practice (*n* = 248)	COR with 95% CI	AOR with 95% CI	*p*‐value
PNC follow‐up	Yes	68		53	Reference	Reference	
	No	81		195	0.32(0.20–0.49)	0.26(0.15–0.46)	**< 0.001**
Number of ANC follow‐ups	Only one	10		12	Reference	Reference	
	Two times	40		72	3.77(0.80–17.65)	5.51(0.95–31.85)	0.06
	Three times	58		87	3.82(0.82–17.70)	4.68(0.79–27.44)	0.09
	Four times	44		51	5.29(1.12–24.93)	5.92(0.99–35.15)	0.051
Place of delivery	Health institution	129		199	Reference	Reference	
	Home delivery	21		48	0.64(0.36–1.13)	1.57(0.75–3.29)	0.23
Maternal education	Up to grade 12	71		178	Reference	Reference	
	Diploma and above	79		69	2.91(1.90–4.45)	3.28(1.88–5.73)	**< 0.001**
Maternal age	< 20years	14		44	Reference	Reference	
	20–24 years	31		61	1.52(0.72–3.19)	0.85(0.35–2.01)	0.71
	25–29 years	54		55	3.14(1.54–6.39)	2.86(1.28–6.42)	**0.01**
	30–34 years	29		35	2.60(1.19–5.66)	4.15(1.58–10.90)	**0.004**
	> 35 years	22		52	1.32(0.60–2.90)	2.19(0.75–6.44)	0.15
Marital status	Unmarried	12		22	Reference	Reference	
	Married	123		185	2.31(0.91–5.86)	0.98(0.34–2.83)	0.98
	Divorced	14		19	2.70(0.86–8.42)	1.73(0.47–6.33)	0.41
	Widowed	12		10	1.83(0.40–8.23)	0.80(0.15–4.35)	0.80
Religion	Muslim	66		95	Reference	Reference	
	Orthodox	75		131	0.83(0.54–1.27)	0.92(0.55–1.52)	0.74
	Protestant	10		20	0.58(0.24–1.39)	0.48(0.17–1.35)	0.17
Family size	1–3	118		179	Reference	Reference	
	4 and more	32		68	0.72(0.44–1.16)	0.39(0.17–0.89)	**0.03**

## 5 Discussion

This study aimed to estimate the prevalence of poor sunlight exposure practices and identify associated factors among mothers in Motta city administration, northwest Ethiopia, in 2023. The study revealed that 38% of mothers exhibited good practices of sunlight exposure, a percentage lower than those reported in previous studies conducted in Yirgalem (54.5%), Debre Birhan town (65.7%), Dessie town (41%), Farta district (54.3%), and Wolkitie University specialized hospital (67.3%) [17, 20, 22, 23, 31]. The higher proportion of poor practice in the current study may be attributed to differences in the study population and timing. Moreover, some of the aforementioned studies were institutional, potentially leading to an overestimation of good practice levels, as mothers with good healthcare‐seeking behavior may also exhibit relatively better sunlight exposure practices. However, the current findings were higher than those reported in Aleta Wondo [14], where only 32% of mothers demonstrated good sunlight exposure practices. This discrepancy could be attributed to sociocultural variations between populations.

In this present investigation, 68.3% of the participants deliberately exposed their infants to sunlight. This percentage was found to be less than the rates reported in previous research conducted in Debre Markos (93%), Debre Birhan (99%), and the study undertaken at Wolkitie University Hospital (73.6%) [17, 31, 32]. The variation in these figures may be attributed to sociocultural diversity and disparities in the utilization of health information among the different study populations.

In this study, 26.6% of mothers, among all those who intentionally exposed their infants to sunlight, initiated the exposure at the age of 15‐20 days after birth. This percentage was slightly higher than the findings in the study conducted in Debre Markos [32], where only 23.4%, and in Farta district [22], where 15.7% of mothers began sunlight exposure at this stage of the baby′s life. However, this result was lower than the findings in the studies conducted in Dejen (52%) and Debre Birhan (85%), where a higher percentage of mothers started exposing their infants to sunlight within 15‐20 days after birth [21, 31]. The variation may be attributed to differences in the health information provided to these populations regarding the appropriate age to commence sunlight exposure.

Among the study participants who intentionally exposed their infants to sunlight, only 28.2% did so daily in this study. This figure was lower compared to findings from Debre Markos (57.9%) and Debre Birhan town (60%) [31, 32]. Additionally, 71.1% of mothers in the current study exposed their infants to sunlight in the morning before 10 AM. This percentage was slightly less than the study conducted in Farta [22], where 73% of mothers exposed their infants during this time. Exposure to sunlight during the morning hours, particularly between 8:00 and 10:00 AM, is crucial for adequate vitamin D synthesis because the Ultraviolet B (UVB) radiation, responsible for triggering vitamin D production in the skin, is present in sufficient quantities during this period. Studies indicate that UVB rays in this time window play a key role in promoting bone health and immune function in infants by facilitating optimal vitamin D synthesis [1, 2]. Furthermore, 54.1% of mothers applied lubricants to their babies while sunning, a proportion higher than the study conducted in Debre Markos [32], where only 43% of respondents applied lubricants during sunlight exposure.

The study revealed that 34.1% of mothers exposed their infants to sunlight for 10‐15 min, and only 17.8% exposed their infants with just a diaper. These figures were notably lower compared to previous studies conducted in different areas [13, 20]. The lower findings in this study could be attributed to poor health information utilization in the study area, and it′s possible that the information regarding the duration of sunning may not be consistent across different regions.

In the current study, the age of the mother was found to be significantly associated with good practices of sunlight exposure for infants. Study participants aged 25–29 years and 30–34 years were three times and four times more likely, respectively, to exhibit good practices in infants′ sunlight exposure compared to younger mothers aged 20 years or younger. This finding is consistent with studies conducted in Wolkitie University Specialized Hospital, Dejen, and Debre Markos [17, 32]. All of these studies indicated that younger mothers were less likely to have good practices in infants′ sunlight exposure compared to older mothers. This observation may be explained by the notion that with increasing maternal age, there is a higher level of awareness regarding sunlight exposure and its importance. Additionally, older mothers may be more prepared for appropriate childbearing, as pregnancies for adolescent mothers may not be planned, potentially affecting their awareness and practices.

In the present study, having a history of postnatal care (PNC) follow‐up was found to have a statistically significant association with good practices in infants′ sunlight exposure. Mothers without a PNC visit were 74% less likely to exhibit good practices in sunlight exposure compared to those with a PNC history. This difference may be attributed to the likelihood that mothers with a PNC history received advice and information about the potential benefits of sunlight exposure for infants.

While institutional delivery was associated with good practices in sunlight exposure in a study conducted in Dejen [21], it did not show significant association in the current study′s multivariable analysis. However, both PNC and institutional delivery could be linked to acquiring proper information and awareness about good practices in sunlight exposure.

The study′s results suggest that mothers with a larger family size were over 60% less likely to adopt good practices in sunlight exposure for their infants compared to mothers with smaller families. This finding is consistent with similar studies conducted in Debre Markos, Mettu, and Debre Birhan town [18, 31, 32]. This suggests that as the size of the family increases, the likelihood of mothers implementing appropriate care practices for their infants, such as sunlight exposure, decreases. This trend may be attributed to the added complexity and increased burden on mothers in larger families, making it more challenging for them to prioritize and maintain optimal care practices for their infants.

The educational level of mothers was found to be significantly associated with good practices in infants′ sunlight exposure. Respondents with a diploma and above level of education were more than three times more likely to exhibit good practices in infant sunlight exposure compared to those with lower levels of education. This association is supported by studies conducted in Debre Markos and Dejen [21, 32]. The rationale behind this finding may be that educated mothers are more informed about the benefits of sunlight exposure and possess a better understanding of the appropriate ways to expose their infants to sunlight. Education appears to play a crucial role in enhancing mothers′ knowledge and practices related to infant care, specifically in the context of sunlight exposure.

### 5.1 Limitation

This study has some limitations. First, the cross‐sectional design limits the ability to establish causal relationships. Second, the data collection was conducted during a specific season, which may affect the generalizability of the findings, as sunlight exposure practices can vary by season. Future studies should consider longitudinal designs that encompass multiple seasons to better understand seasonal variations in infant sunlight exposure practices.

## 6 Conclusion

The level of good practice in infants′ sunlight exposure was found to be very low in the study area, with two‐thirds of the study participants not appropriately exposing their infants to sunlight. Younger maternal age, larger family size, poor maternal education, and lack of postnatal follow‐up were identified as statistically significant factors affecting mothers′ good practices in sunlight exposure. Strategic interventions should be implemented to minimize early‐age pregnancies, expand family planning initiatives, increase utilization of postnatal care services, and improve access to women′s education. Health education on the importance and correct timing of infant sunlight exposure should be integrated into postnatal care services, with particular emphasis on reaching younger and less‐educated mothers. Improving maternal knowledge through community‐based education and counseling can help ensure that caregivers understand when and how to safely expose their infants to sunlight, particularly during the early morning hours when ultraviolet B (UVB) rays are effective for vitamin D synthesis but less harmful to the skin.

## Author Contributions

**Tesfaye Shumet Mekonnen:** conceptualization, data curation, formal analysis, funding acquisition, methodology, investigation, project administration, resources, software, writing – original draft, and writing – review and editing. **Eniyew Talie Fenta:** methodology, project administration, resources, software, and writing – review and editing. **Anley Shiferaw Enawgaw:** methodology, project administration, visualization, supervision, resources, software, and writing – review and editing. **Fasikaw Kebede Bizuneh:** methodology, investigation, project administration, validation, resources, supervision, software, and writing – review and editing. **Amare Mebrat Delie:** conceptualization, data curation, formal analysis, funding acquisition, methodology, investigation, project administration, resources, software, writing—original draft, and writing – review and editing. All authors have read and approved the final version of the manuscript. Tesfaye Shumet Mekonnen had full access to all of the data in this study and takes complete responsibility for the integrity of the data and the accuracy of the data analysis.

## Ethics Statement

The study was conducted following ethical guidelines, including the Declaration of Helsinki. The research procedures and objectives were thoroughly reviewed by the institutional review board of Woldia University College of Health Sciences. Ethical approval was granted on June 25, 2024, with reference number RCS, TT & UIL; 0025/2016. The study was conducted while the corresponding author was affiliated with Woldia University, which granted ethical approval for this study. The current affiliation with Debre Markos University reflects a subsequent institutional transfer. Following approval, an official letter was submitted to both the town health office and the administrative body. Informed consent was secured from every study participant, accompanied by a clear explanation of the study′s purpose and assurance of confidentiality. Participants were informed of their full right to choose not to partake in the study or to discontinue participation at any point during the interview.

## Conflicts of Interest

The authors declare no conflicts of interest.

## Transparency Statement

The lead author Tesfaye Shumet Mekonnen affirms that this manuscript is an honest, accurate, and transparent account of the study being reported; that no important aspects of the study have been omitted; and that any discrepancies from the study as planned (and, if relevant, registered) have been explained.

## Data Availability

The data that support the findings of this study are available from the corresponding author upon reasonable request.

## References

[1] A. Jindal , A. Gupta , K. Vinay , and A. Bishnoi , “Sun Exposure In Children: Balancing the Benefits and Harms,” Indian Dermatology Online Journal 11, no. 1 (2020): 94–98. 32055519 10.4103/idoj.IDOJ_206_19 PMC7001416

[2] N. M. M. Mansibang , M. G. Y. Yu , C. A. Jimeno , and F. L. Lantion‐Ang , “Association of Sunlight Exposure With 25‐hydroxyvitamin D Levels Among Working Urban Adult Filipinos,” Osteoporosis and Sarcopenia 6, no. 3 (2020): 133–138. 33102807 10.1016/j.afos.2020.08.006 PMC7573503

[3] A. Y. Kurihayashi , R. A. Augusto , F. M. D. Escaldelai , and L. A. Martini , “Estado Nutricional De Vitaminas A E D Em Crianças Participantes De Programa De Suplementação Alimentar,” Cadernos de Saúde Pública 31, no. 3 (2015): 531–542. 25859720 10.1590/0102-311x00082814

[4] S. Agarwal , O. Kovilam , and D. K. Agrawal , “Vitamin D and Its Impact on Maternal‐Fetal Outcomes in Pregnancy: A Critical Review,” Critical Reviews in Food Science and Nutrition 58, no. 5 (2018): 755–769. 27558700 10.1080/10408398.2016.1220915 PMC6056893

[5] A. Asyary and M. Veruswati , “Sunlight Exposure Increased Covid‐19 Recovery Rates: A Study in the Central Pandemic Area of Indonesia,” Science of the Total Environment 729 (2020): 139016. 32361458 10.1016/j.scitotenv.2020.139016 PMC7184988

[6] H. B. Kim and J. H. Kim , “Sunlight Exposure in Association With Risk of Lymphoid Malignancy: A Meta‐Analysis of Observational Studies,” CancEr Causes & Control: CCC 32, no. 5 (2021): 441–457. 33606147 10.1007/s10552-021-01404-6

[7] S. T. Kent , M. Cushman , G. Howard , et al., “Sunlight Exposure and Cardiovascular Risk Factors in the REGARDS Study: A Cross‐Sectional Split‐Sample Analysis,” BMC Neurology 14 (2014): 133. 24946776 10.1186/1471-2377-14-133 PMC4075775

[8] M. G. Yu , N. Castillo‐Carandang , M. E. G. Sison , et al., “Attitudes, Behaviors and Beliefs of Urban Adult Filipinos on Sunlight Exposure: A Qualitative Study,” Journal of the ASEAN Federation of Endocrine Societies 33, no. 1 (2018): 37–43. 33442109 10.15605/jafes.033.01.06 PMC7784093

[9] S. Boonrusmee , S. Kasemsripitak , T. Navykarn , and S. Jaruratanasirikul , “Association Between Anaemia and Vitamin D Insufficiency Among 6‐ to 12‐month‐old Infants: Implications for Clinical Practice,” Family Practice 41, no. 3 (2023): 305–311. 10.1093/fampra/cmad033 37014969

[10] P. Meena , A. Dabas , D. Shah , R. K. Malhotra , S. V. Madhu , and P. Gupta , “Sunlight Exposure and Vitamin D Status in Breastfed Infants,” Indian Pediatrics 54, no. 2 (2017): 105–111. 28031546 10.1007/s13312-017-1010-9

[11] H. Zhang , S. Liu , Y. Si , et al., “Natural Sunlight Plus Vitamin D Supplementation Ameliorate Delayed Early Motor Development in Newborn Infants From Maternal Perinatal Depression,” Journal of Affective Disorders 257 (2019): 241–249. 31301627 10.1016/j.jad.2019.07.010

[12] H. Kanemura , K. Hatakeyama , F. Sano , H. Yagasaki , K. Sugita , and M. Aihara , “Effect of Sunlight Exposure on Bone Mineral Density in Children With Severe Disability,” Neuropediatrics 47, no. 4 (2016): 233–237. 27227999 10.1055/s-0036-1584083

[13] Y. G. Ashebir , G. T. Sebsibe , D. Gela , and M. A. Kebede , “Attitudes of Mothers Attending Public Hospitals in Addis Ababa, Ethiopia, to Neonatal Sunlight Exposure: A Cross‐Sectional Study,” BMJ Paediatrics Open 6, no. 1 (2022): e001554. 36645760 10.1136/bmjpo-2022-001554 PMC9454020

[14] A. Bedaso , M. Gebrie , B. Deribe , M. Ayalew , and B. Duko , “Knowledge and Practice on Adequate Sunlight Exposure of Infants Among Mothers Attending Epi Unit of Aleta Wondo Health Center, SNNPR, Ethiopia,” BMC Research Notes 12, no. 1 (2019): 183. 30922365 10.1186/s13104-019-4221-4 PMC6440125

[15] A. K. Amegah , M. Nsoh , G. Ashley‐Amegah , and J. Anaman‐Togbor , “What Factors Influences Dietary and Non‐Dietary Vitamin D Intake Among Pregnant Women in an African Population?,” Nutrition 50 (2018): 36–44. 29522981 10.1016/j.nut.2017.11.003

[16] E. A. Lake , B. W. Demissie , N. A. Gebeyehu , et al., “Knowledge and Practice of Mothers Towards Sunshine Exposure of Their Children in Ethiopia: A Systematic Review and Meta‐Analysis,” BMC Pediatrics 22, no. 1 (2022): 213. 35436897 10.1186/s12887-022-03281-7 PMC9014620

[17] G. T. Mengistu , A. B. Terefe , T. G. Gudeta , and B. K. Mengistu , “Factors Associated With Infants′ Sunlight Exposure Among Mothers Attending the EPI Unit of Wolkite University Specialized Hospital,” PLoS One 17, no. 11 (2022): e0277349. 36395250 10.1371/journal.pone.0277349 PMC9671437

[18] A. Tadesse , S. Yeshanew , and A. G. Gedefa , “Knowledge and Practice of Infant Exposure to Sunlight Among Mothers in the Rural Villages of Mettu District, Southwest Ethiopia,” Frontiers in Public Health 11 (2023): 1166976. 37529425 10.3389/fpubh.2023.1166976 PMC10390293

[19] A. Y. M. Leung , M. K. T. Cheung , and I. Chi , “Supplementing Vitamin D Through Sunlight: Associating Health Literacy With Sunlight Exposure Behavior,” Archives of Gerontology and Geriatrics 60, no. 1 (2015): 134–141. 25456887 10.1016/j.archger.2014.10.005

[20] A. S. Bezabih , D. Eshetu , N. Yohanis , and A. T. Hirigo , “Knowledge and Practice of Infants Exposure to Sunlight Among Lactating Mothers Attending at Yirgalem Hospital, Sidama Regional State,” Clinical Medicine Insights: Pediatrics 15 (2021): 11795565211041348. 34552362 10.1177/11795565211041348 PMC8450552

[21] A. Bekalu , A. Molla , B. Asmare , Y. Hune , and H. Temesgen , “Practice of Sunlight Exposure of Infants and Associated Factors Among Infant Coupled Mothers at Dejen District, Amhara Region, Northwest Ethiopia 2021,” Nutrition and Metabolic Insights 15 (2022): 11786388221106983. 35799616 10.1177/11786388221106983 PMC9253983

[22] H. Gedamu and Y. Tafere , “Assessment of Knowledge, Attitude, and Practice of Sunlight Exposure of Infants Among Mothers Attending in Governmental Health Facilities in Farta District, South Gondar Zone, North West Ethiopia, 2018,” International Journal of Reproductive Medicine 2019 (2019): 1–7. 10.1155/2019/2638190 PMC6791214 31662960

[23] D. Goshiye , G. Biset , Z. Abegaz , E. Birrie , and S. Gedamu , “Knowledge, Practice, and Factors Affecting Sunlight Exposure of Infants Among Mothers at Governmental Health Facilities in Dessie Town, Ethiopia, 2021,” Clinical Medicine Insights: Pediatrics 17 (2023): 11795565221148329. 36686984 10.1177/11795565221148329 PMC9850123

[24] H. Balogun , J. J. K. Jaakkola , and A. K. Amegah , “Association of Sunlight Exposure and Consumption of Vitamin D‐Rich Foods During Pregnancy With Adverse Birth Outcomes in an African Population,” Journal of Tropical Pediatrics 65, no. 6 (2019): 526–536. 30690592 10.1093/tropej/fmz001

[25] C. Ilmiawati , A. Oviana , A. Friadi , and M. Reza , “Sunlight Exposed Body Surface Area Is Associated With Serum 25‐hydroxyvitamin D (25(OH)D) Level in Pregnant Minangkabau Women, Indonesia,” BMC nutrition 6 (2020): 18. 32467767 10.1186/s40795-020-00342-x PMC7232832

[26] D. H. Koh , J. H. Park , S. G. Lee , et al., “Assessment of Sunlight Exposure Across Industries and Occupations Using Blood Vitamin D as a Biomarker,” Journal of Occupational Health 64, no. 1 (2022): e12318. 35152501 10.1002/1348-9585.12318 PMC8841173

[27] K. T. Kanatani , T. Nakayama , Y. Adachi , et al., “High Frequency of Vitamin D Deficiency in Current Pregnant Japanese Women Associated With UV Avoidance and Hypo‐Vitamin D Diet,” PLoS One 14, no. 3 (2019): e0213264. 30830935 10.1371/journal.pone.0213264 PMC6398852

[28] M. A. Abdulrahman , S. Y. Alkass , and N. I. Mohammed , “Total and Free Vitamin D Status Among Apparently Healthy Adults Living in Duhok Governorate,” Scientific Reports 12, no. 1 (2022): 1778. 35110608 10.1038/s41598-022-05775-x PMC8810798

[29] B. Abebaw and D. Damtie , “Pneumonia Prevalence and Associated Risk Factors Among Under‐Five Children in Goncha Siso Enesie District, Northwest Ethiopia,” Advances in Public Health 2022 (2022): 1–8.

[30] A. Bekalu , A. Molla , B. Asmare , Y. Hune , and H. Temesgen , “Practice of Sunlight Exposure of Infants and Associated Factors Among Infant Coupled Mothers at Dejen District, Amhara Region, Northwest Ethiopia 2021,” Nutrition and Metabolic Insights 15 (2022): 11786388221106983. 35799616 10.1177/11786388221106983 PMC9253983

[31] W. Z. Teklehaimanot , L. D. Kitawu , T. Tesfaye , et al., “Assessment of Practice and Factors Associated With Sunlight Exposure of Infants Among Mothers in Debre Berhan Town, North Shewa Zone, Amhara Region, Ethiopia,” Pediatric Health, Medicine and Therapeutics 12 (2021): 507–517. 34795548 10.2147/PHMT.S330896 PMC8593595

[32] A. Abate , “Assessment of Practice and Factors Affecting Sunlight Exposure of Infants Among Mothers Attending Governmental Health Facilities in Debre Markos Town, East Gojjam, Ethiopia, 2015,” American Journal Of Nursing Science 5 (2016): 30–36.

